# Increased NKX6.1 expression and decreased ARX expression in alpha cells accompany reduced beta-cell volume in human subjects

**DOI:** 10.1038/s41598-021-97235-1

**Published:** 2021-09-07

**Authors:** Yukari Fujita, Junji Kozawa, Kenji Fukui, Hiromi Iwahashi, Hidetoshi Eguchi, Iichiro Shimomura

**Affiliations:** 1 Departments of Metabolic Medicine, Graduate School of Medicine, Osaka University, 2-2-B5 Yamadaoka, Suita, 565-0871 Japan; 2 Community Medicine, Graduate School of Medicine, Osaka University, Suita, Japan; 3 Diabetes Care Medicine, Graduate School of Medicine, Osaka University, Suita, Japan; 4 Gastroenterological Surgery, Graduate School of Medicine, Osaka University, Suita, Japan

**Keywords:** Cell biology, Health care

## Abstract

Pancreatic islet cells have plasticity, such as the abilities to dedifferentiate and transdifferentiate. Islet cell conversion to other characteristic cell is largely determined by transcription factors, but significance of expression patterns of these transcription factors in human islet cells remained unclear. Here, we present the NKX6.1-positive ratio of glucagon-positive cells (NKX6.1^+^/GCG^+^ ratio) and the ARX-negative ratio of glucagon-positive cells (ARX^−^/GCG^+^ ratio) in 34 patients who were not administered antidiabetic agents. Both of NKX6.1^+^/GCG^+^ ratio and ARX^−^/GCG^+^ ratio negatively associated with relative beta cell area. And these ratios did not have significant correlation with other parameters including age, body mass index, hemoglobin A1c, fasting plasma glucose level or relative alpha-cell area. Our data demonstrate that these expression ratios of transcription factors in glucagon-positive cells closely correlate with the reduction of beta-cell volume in human pancreas.

## Introduction

Beta-cell volume is decreased in type 2 diabetes patients^1,2^, and one of the mechanisms behind is beta-cell apoptosis^2^, but the remaining mechanism has not been elucidated. It has been suggested that pancreatic islet cells have plasticity, such as the abilities to dedifferentiate and transdifferentiate. Then, another mechanism may be due to beta-cell dedifferentiation^3^.

It is well known that islet cells convert to the cells with other characteristics under various artificial conditions. Epigenomic manipulation is thought to provide a path to alpha- to beta-cell reprogramming^4^, and searches for materials that promote alpha- to beta-cell conversion are also progressing^5–7^. Genetic manipulations of transcription factors are also often conducted to induce transdifferentiation of islet cells. Beta cells acquire alpha- and PP-cell phenotype by misexpression of Arx^8^. In an animal model or a cell line, inactivation of aristaless-related homeobox (Arx) gene in mouse alpha cells^9^, or Pax4 gene transfer into αTC1.9 cells results in transdifferentiation to beta-like cells^10^. Lineage tracing and single-cell RNA sequencing revealed that both of DNA methyltransferase 1 and Arx loss leads to extensive alpha-cell conversion into progeny resembling native beta cells^11^. Infusion of adeno-associated virus carrying Pdx1 and MafA expression cassettes through the pancreatic duct can reprogram alpha cells into functional beta cells and normalize blood glucose level in both beta cell-toxin-induced diabetic mice and in autoimmune non-obese diabetic (NOD) mice^12^. Conversely, when transdifferentiation occurs, the expression patterns of transcription factors change in islet cells. Extreme loss of beta cells in mouse induces the conversion of alpha cells to beta cells, in which beta-cell-specific transcription factors such as pancreatic duodenal homeobox factor-1 (Pdx-1) and NK homeobox 6.1 (Nkx6.1) are expressed in alpha cells^13^. All these phenomena are observed under artificial conditions. On the other hand, it is assumed that islet-cell conversion also occurs in physiological conditions. A study using lineage tracing in mouse revealed that immature beta cells present at islet periphery are in an intermediate transdifferentiation stage between alpha and beta cells^14^. In human isolated islets, it is revealed that mature human beta cells convert to glucagon producing cells just by culturing islets^15^. We recently identified beta cells that expressed ARX or that did not express PDX-1, and alpha cells that did not express ARX or that express PDX-1 widely ranging from normal glucose tolerance to diabetic glucose tolerance stages, using human pancreatic fresh tissue samples obtained by pancreatectomy^16^. However, the factors that promote the changes of these expression patterns of transcription factors in physiological condition in humans have not been elucidated.

The purpose of this study is to investigate the factors related to transdifferentiation by evaluating the proportions of cells positive for NKX6.1 and negative for ARX. The reason that we used these transcription factors was that NKX6.1-positive progenitors are used for marking pancreatic beta cells during human beta-cell development^17^, and ARX is one of the most important transcription factors that participates in the differentiation of alpha cells^18^, among human alpha cells and their correlations with clinical and histological parameters. The included patients had not been administered antidiabetic agents because insulin therapy^19^, GLP-1 (glucagon-like peptide-1) receptor agonist^20,21^ and DPP-4 (dipeptidyl peptidase-4) inhibitor^22^ are thought to affect islet-cell transdifferentiation.

### Conference presentation

Parts of this study were presented at the 64nd Annual Meeting of the Japan Diabetes Society, online, 20–22 May, 2021; the 93rd Annual Congress of the Japan Endocrine Society, online, 20 July to 31 August, 2020; the 62nd Annual Meeting of the Japan Diabetes Society, Sendai, Japan, 23–25 May, 2019; and the 61st Annual Meeting of the Japan Diabetes Society, Tokyo, Japan, 24–26 May, 2018.

### Ethical considerations

All procedures followed in this study were in accordance with the ethical standards of the responsible committee on human experimentation (institutional and national) and with the Declaration of Helsinki 1975, as revised in 2013. Informed consent was obtained from all subjects who participated in the study.

## Result

### Clinical characteristics and histological data

Table 1 lists the characteristics of the patients. The patients were classified as having normal glucose tolerance (NGT) (n = 14), impaired glucose tolerance (IGT) (n = 10), or diabetes mellitus (DM) (n = 10) on the basis of a 75-g oral glucose tolerance test (OGTT). The main primary diseases were pancreatic cancer (n = 12) and cystic lesions of the pancreas (n = 12). The operative procedures were pancreatoduodenectomy (n = 23), distal pancreatectomy (n = 10), and total pancreatectomy (n = 1). Ten patients had been treated with anticancer agents before surgery. The average age was 65 ± 11 years, BMI was 21.6 ± 2.7 kg/m^2^, HbA1c was 5.7 ± 0.6% (38 ± 7 mmol/mol), fasting plasma glucose (FPG) was 5.4 ± 0.6 mmol/L, fasting C-peptide immunoreactivity (F-CPR) was 0.52 ± 0.20 nmol/L, and increment of C-peptide by glucagon test (ΔC-peptide) was 0.99 ± 0.38 nmol/L. HbA1c in DM patient was significantly higher than NGT (p < 0.01) and IGT (p < 0.05). There were no significant differences among the three groups in other clinical parameters. The relative alpha-cell area was 0.16 ± 0.12%, the relative beta-cell area was 0.94 ± 0.44%, the alpha- to beta-cell area ratio (α/β) was 0.18 ± 0.12, and the INS^+^/GCG^+^ ratio was 0.99 ± 1.00%. The relative beta-cell area decreased in patients with type 2 diabetes, although there was no statistically significant difference. There were not significant differences among the three groups also in all other histological parameters.

**Table 1:** Patient clinical characteristics and data.

	Total	NGT	IGT	DM
N (male/female)	34 (21/13)	14 (9/5)	10 (5/5)	10 (7/3)
**Clinical diagnosis**				
Pancreatic cancer	12 (35%)	4	5	3
Cystic lesions of the pancreas	12 (35%)	6	3	3
Cholangiocarcinoma	3 (9%)	1	2	0
Tumor of the ampulla of Vater	3 (9%)	1	0	2
Hepatocellular carcinoma	1 (3%)	1	0	0
Cholangitis	1 (3%)	1	0	0
Chronic pancreatitis	1 (3%)	0	0	1
Pancreatic metastasis of renal cell carcinoma	1 (3%)	0	0	1
Operative procedure (PD/DP/total)	23/10/1	12/2/0	5/4/1	6/4/0
Preoperative anticancer agents (yes/no)	10/24	4/10	4/6	2/8
Age (years)	65 ± 11	64 ± 11	62 ± 15	68 ± 6
BMI (kg/m^2^)	21.6 ± 2.7	21.4 ± 3.0	20.9 ± 1.8	22.4 ± 3.1
HbA_1c_ (mmol/mol, %)	38 ± 7, 5.7 ± 0.6	35 ± 6, 5.3 ± 0.6	37 ± 6, 5.6 ± 0.5	45 ± 5, 6.2 ± 0.4**†
FPG (mmol/L, mg/dL)	5.4 ± 0.5, 97 ± 9	5.4 ± 0.4, 96 ± 2	5.2 ± 0.5, 93 ± 3	5.6 ± 0.6, 101 ± 3
F-CPR (nmol/L, ng/mL)	0.52 ± 0.20, 1.6 ± 0.6	0.56 ± 0.22, 1.7 ± 0.7	0.48 ± 0.15, 1.5 ± 0.5	0.49 ± 0.23, 1.5 ± 0.7
ΔC-peptide (nmol/L, ng/mL) (n = 19)	0.99 ± 0.38, 3.0 ± 1.2	1.09 ± 0.40, 3.3 ± 1.2 (n = 10)	0.98 ± 0.34, 3.0 ± 1.0 (n = 4)	0.81 ± 0.37, 2.5 ± 1.1 (n = 5)
Relative alpha-cell area (%)	0.16 ± 0.12	0.17 ± 0.09	0.16 ± 0.11	0.17 ± 0.16
Relative beta-cell area (%)	0.94 ± 0.44	1.02 ± 0.45	1.01 ± 0.40	0.76 ± 0.44
Alpha- to beta-cell area ratio	0.18 ± 0.12	0.17 ± 0.10	0.17 ± 0.12	0.19 ± 0.14
INS^+^/GCG^+^ ratio (%)	0.99 ± 1.00	0.83 ± 0.50	1.10 ± 1.48	1.08 ± 1.02

*NGT* normal **glucose** tolerance, *IGT* impaired **glucose** tolerance, *DM* diabetes mellitus, *PD* pancreaticoduodenectomy, *DP* distal pancreatectomy, *FPG* fasting plasma glucose, *F-CPR* fasting C-peptide immunoreactivity, *ΔC-peptide* increment of C-peptide immunoreactivity level by glucagon test, *INS*^*+*^*/GCG*^*+*^* ratio* insulin-positive ratio of glucagon-positive cells, *NKX6.1*^*+*^*/GCG*^*+*^* ratio* NKX6.1-positive ratio of glucagon-positive cells, *ARX*^*−*^*/GCG*^*+*^* ratio* ARX-negative ratio of glucagon-positive cells, *NKX6.1* NK6 homeobox 1, *ARX* aristaless-related homeobox. Mean ± SD. One-way analysis of variance followed by a post hoc Tukey–Kramer analysis. **p < 0.01 vs NGT, †p < 0.05 vs IGT.

### NKX-positive and ARX-negative ratio of glucagon-positive cells

We analyzed 694 ± 578 glucagon-positive cells to evaluate the NKX6.1-positive ratios and 628 ± 468 glucagon-positive cells per patient to evaluate the ARX-negative ratios. The average NKX6.1^+^/GCG^+^ ratios were 3.2 ± 1.5%, 4.2 ± 2.3%, and 4.6 ± 3.2% in the NGT, IGT, and DM groups, respectively, which did not differ significantly (p = 0.312). The average ARX^−^/GCG^+^ ratios were 18.7 ± 5.9%, 24.9 ± 2.6%, and 17.8 ± 2.6% in the NGT, IGT, and DM groups, respectively, which were also not significantly different (p = 0.113).

### The NKX6.1-positive ratio and clinical or histological parameters

**Figure 1:**
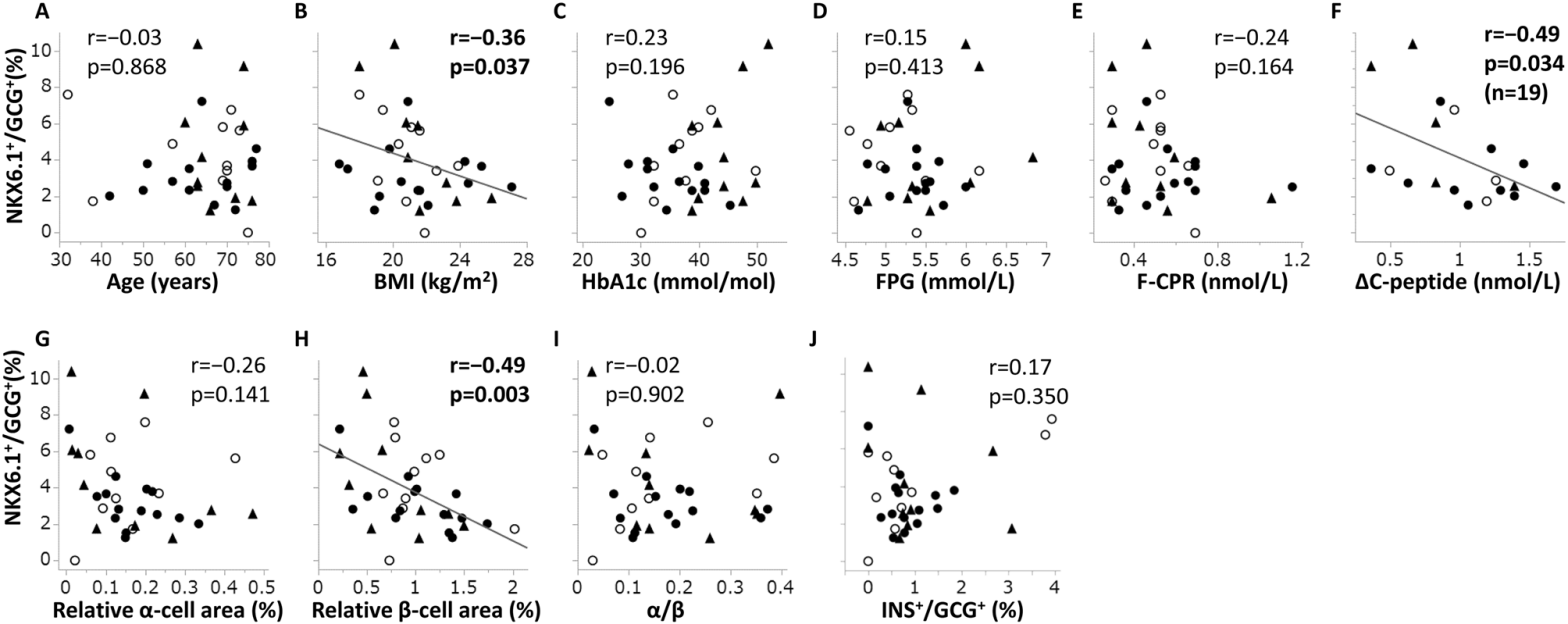
Correlation coefficients between the NKX6.1-positive ratio of glucagon-positive cells and various parameters. NKX6.1-positive ratio of glucagon-positive cells (NKX6.1^+^/GCG^+^ ratio) and age (**A**), BMI (**B**), HbA1c (**C**), FPG (**D**), F-CPR (**E**), ΔC-peptide (**F**), relative alpha-cell area (**G**), relative beta-cell area (H), α/β (**I**), and INS^+^/GCG^+^ ratio (**J**). Closed circles (●), normal glucose tolerance; open circles (○), impaired glucose tolerance; closed triangles (▲), diabetes; *NKX6.1* NK homeobox 6.1, *FPG* fasting plasma glucose, *F-CPR* fasting C-peptide immunoreactivity, *ΔC-peptide* increment of C-peptide by glucagon test, *α/β* alpha- to beta-cell area ratio, *r* correlation coefficient, *p* p value.

Figure 1 shows regression analyses between the NKX6.1^+^/GCG^+^ ratio and various clinical or histological parameters. This ratio had significant negative correlations with BMI (r =  − 0.36, p = 0.037), ΔC-peptide (r =  − 0.49, p = 0.034), and relative beta-cell area (r =  − 0.49, p = 0.003) (Fig. 1B,F,H). However, this ratio did not have a significant correlation with age (Fig. 1A), HbA1c (Fig. 1C), FPG (Fig. 1D), F-CPR (Fig. 1E), relative alpha-cell area (Fig. 1G), α/β (Fig. 1I), or INS^+^/GCG^+^ ratio (Fig. 1J). In multiple regression analysis performed with the variables that correlated significantly with NKX6.1^+^/GCG^+^ ratio, the relative beta-cell area (F-value = 8.07, p = 0.012) was the only independent and significant determinant of NKX6.1^+^/GCG^+^ ratio, while BMI (F-value = 0.75, p = 0.399) and ΔC-peptide (F-value = 0.01, p = 0.933) were not.

### The ARX-negative ratio and clinical or histological parameters

**Figure 2:**
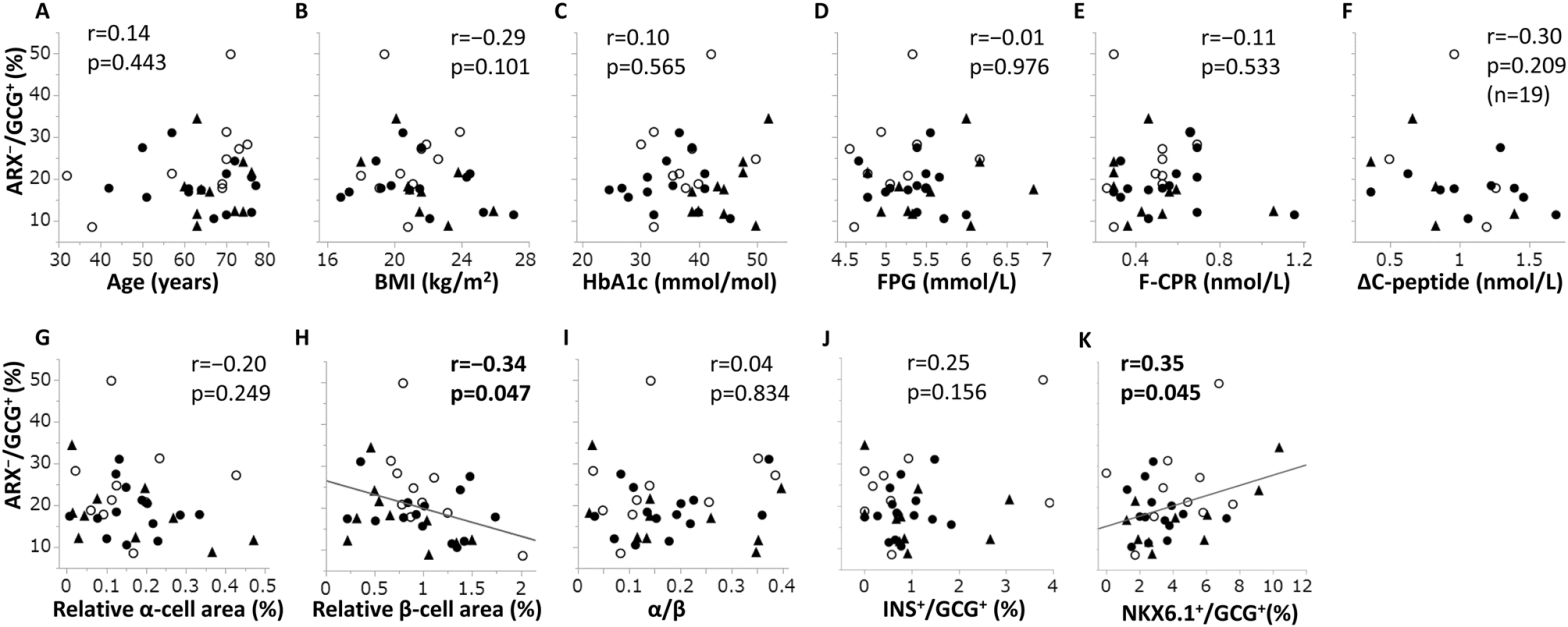
Correlation coefficients between the ARX-negative ratio of glucagon-positive cells and various parameters. ARX-negative ratio of glucagon-positive cells (ARX^−^/GCG^+^ ratio) and age (**A**), BMI (**B**), HbA1c (**C**), FPG (**D**), F-CPR (**E**), ΔC-peptide (**F**), relative alpha-cell area (**G**), relative beta-cell area (**H**), α/β (**I**), INS^+^/GCG^+^ ratio (**J**), and NKX6.1^+^/GCG^+^ ratio (**K**). Closed circles (●), normal glucose tolerance; open circles (○), impaired glucose tolerance; closed triangles (▲), diabetes; *ARX* aristaless-related homeobox, *FPG* fasting plasma glucose, *F-CPR* fasting C-peptide immunoreactivity, *ΔC-peptide* increment of C-peptide by glucagon test, *α/β* alpha- to beta-cell area ratio, *NKX6.1* NK homeobox 6.1. Pearson's correlation coefficient was used. r, correlation coefficient; p, p value.

Figure 2 shows regression analyses between the ARX^−^/GCG^+^ ratio and various clinical or histological parameters. This ratio had a significant negative correlation only with the relative beta-cell area (r =  − 0.34, p = 0.047) (Fig. 2H). This ratio did not have a significant correlation with age (Fig. 2A), BMI (Fig. 2B), HbA1c (Fig. 2C), FPG (Fig. 2D), F-CPR (Fig. 2E), ΔC-peptide (Fig. 2F), relative alpha-cell area (Fig. 2G), α/β (Fig. 2I), or INS^+^/GCG^+^ ratio (Fig. 2J). The ARX^−^/GCG^+^ ratio had a significant positive correlation with the NKX6.1^+^/GCG^+^ ratio (r = 0.35, p = 0.045) (Fig. 2K).

### The NKX6.1-positive/ARX-negative ratio and relative beta-cell area in each NGT, IGT and DM group and in the combination of IGT and DM groups

We verified the correlation between the NKX6.1^+^/GCG^+^ ratio or the ARX^−^/GCG^+^ ratio of glucagon-positive cells and the relative beta-cell area in each NGT, IGT and DM group and in the combination of IGT and DM (IGT + DM) groups. The NKX6.1^+^/GCG^+^ ratio in NGT had a significant negative correlation with the relative beta-cell area (r =  − 0.62, p = 0.017) (Suppl. Fig. A), and the ratios in DM (r =  − 0.60, p = 0.068) and IGT + DM (r =  − 0.44, p = 0.054) had moderate negative correlations although they were not statistically significant (Suppl. Fig. C and D). The ARX^−^/GCG^+^ ratios had negative correlations in IGT (r =  − 0.63, p = 0.058), DM (r =  − 0.50, p = 0.145) and IGT + DM (r =  − 0.38, p = 0.098) although they were not statistically significant (Suppl. Fig. F, G and H). There were no correlations in NKX6.1^+^/GCG^+^ ratio in IGT (Suppl. Fig. B) and ARX^−^/GCG^+^ ratio in NGT (Supple Fig. E).

### Representative images of islet immunostaining

**Figure 3:**
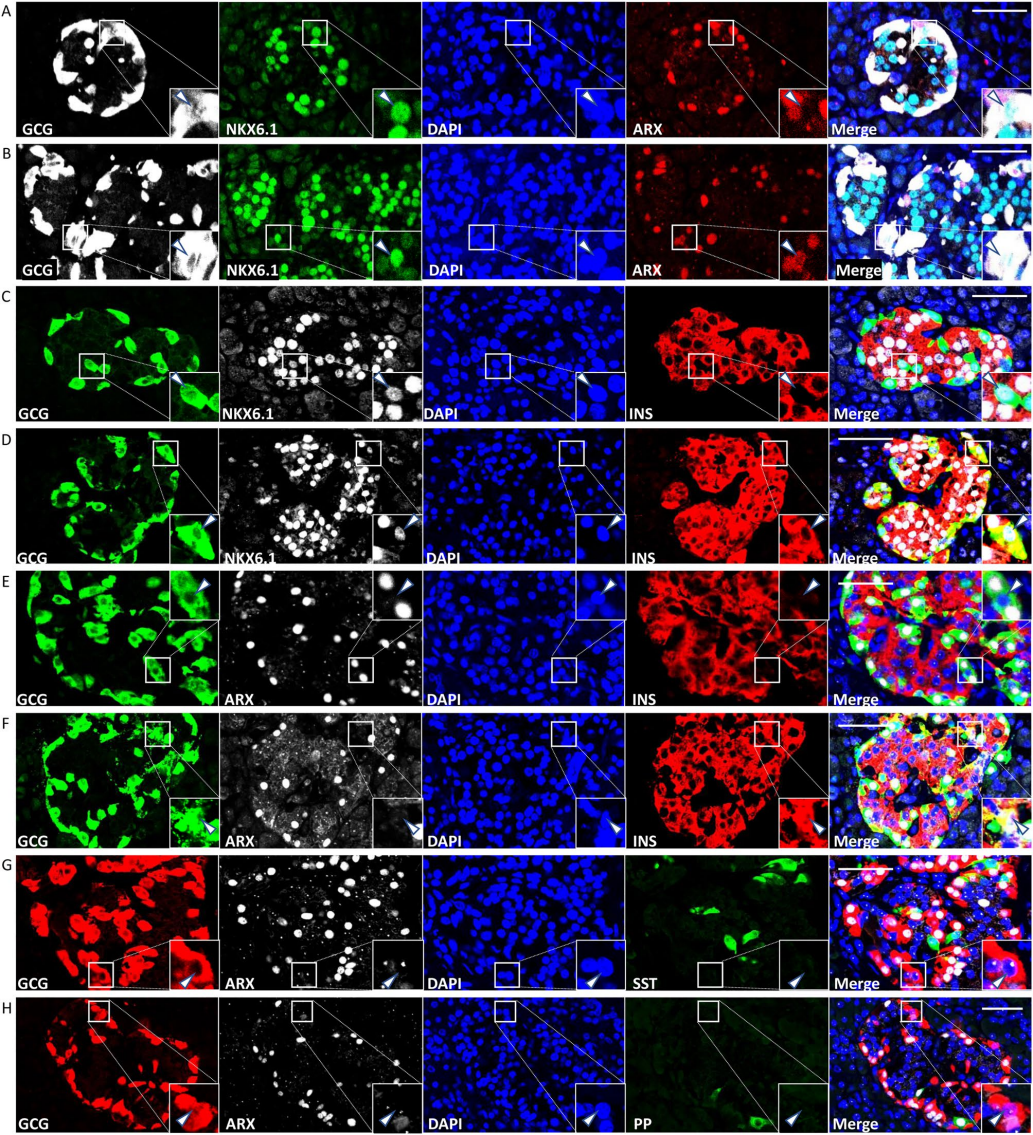
Representative images of islet immunostaining of an NGT female in her 50 s photographed under a laser scanning confocal microscope. (**A**) Triple immunostaining of GCG (white), NKX6.1 (green), and ARX (red). White arrowhead shows ARX-negative NKX6.1^+^/GCG^+^ cell. (**B**) Triple immunostaining of GCG (white), NKX6.1 (green), and ARX (red). White arrowhead shows ARX-positive NKX6.1^+^/GCG^+^ cell. (**C**) Triple immunostaining of GCG (green), NKX6.1 (white), and INS (red). White arrowhead shows INS-negative NKX6.1^+^/GCG^+^ cell. (**D**) Triple immunostaining of GCG (green), NKX6.1 (white), and INS (red). White arrowhead shows INS-positive NKX6.1^+^/GCG^+^ cell. (**E**) Triple immunostaining of GCG (green), ARX (white), and INS (red). White arrowhead shows INS-negative ARX^−^/GCG^+^ cell. (**F**) Triple immunostaining of GCG (green), ARX (white), and INS (red). White arrowhead shows INS-positive ARX^−^/GCG^+^ cell. (**G**) Triple immunostaining of GCG (red), ARX (white), and SST (green). White arrowhead shows SST-negative ARX^−^/GCG^+^ cell. (**H**) Triple immunostaining of GCG (red), ARX (white), and PP (green). White arrowhead shows PP-negative ARX^−^/GCG^+^ cell. *GCG* glucagon, *NKX6.1* NK homeobox 6.1, *INS* insulin, *ARX* aristaless-related homeobox, *SST* somatostatin, *PP* pancreatic polypeptide, *DAPI* 4′,6-diamidino-2-phenylindole. Bars = 50 µm.

Figure 3 shows images of immunostaining photographed using a laser scanning confocal microscope (FV-1200; Olympus, Tokyo, Japan). Figure 3A–D show NKX6.1^+^/GCG^+^ cells. Some NKX6.1^+^/GCG^+^ cells did not express ARX (Fig. 3A), while others did (Fig. 3B); meanwhile, some NKX6.1^+^/GCG^+^ cells did not express insulin (Fig. 3C), while others did (Fig. 3D). Figure 2E–H show ARX^−^/GCG^+^ cells. Some ARX^−^/GCG^+^ cells did not express insulin (Fig. 3E), while others did (Fig. 3F). The ARX^−^/GCG^+^ cells did not express somatostatin (Fig. 3G) or pancreatic polypeptide (Fig. 3H).

## Discussion

We revealed that the NKX6.1-positive ratio and ARX-negative ratio of alpha cells increased with a reduction of beta-cell volume in human pancreas. Moreover, we confirmed that there was a significant positive correlation between NKX6.1-positive ratio and ARX-negative ratio. These data strongly suggest that ARX loss and NKX6.1 expression might occur in parallel. Because neither HbA1c nor FPG level had any correlation with the NKX6.1-positive ratio or ARX-negative ratio, only the reduction of beta-cell volume may correlate with the change of expression patterns of these transcription factors.

In type 2 diabetes patients, the expressions of PDX-1, MAFA, or NKX6.1 are reduced in beta cells^23^, and the expression of NKX6.1 is increased in glucagon-positive cells^24^. In type 1 diabetes patients, alpha cells have reduced expression of ARX and increased expression of NKX6.1^25^. According to these reports, transdifferentiation of islet cells might also occur in diabetes in humans, probably most of whom were administered antidiabetic agents. In these reports, it has remained unclear which diabetes-related factor, such as hyperglycemia, the reduction of insulin level, or antidiabetic agents, affects these expression changes. This study, which excluded cases under treatment with hypoglycemic agents, first revealed that the expression pattern changes of transcription factors were associated with beta-cell volume.

Insulin and glucagon co-expressing cells are often found in the pancreas of embryonic pancreas^26^ and appears in the course of transdifferentiation^5,13^, and they are considered as differentiating islet cells. We found that insulin and glucagon co-expressing cells have various expression patterns of transcription factors as well as our previous report^16^. In this study, there were a few INS^+^/GCG^+^ cells, but the INS^+^/GCG^+^ ratio did not have a significant correlation with NKX6.1^+^/GCG^+^ ratio or ARX^-^/GCG^+^ ratio. The reason seems to be that insulin and glucagon co-expressing cells may contain cells under neoplasia, dedifferentiation and transdifferentiation into any type of islet cells.

There are some reports regarding the processes in which these beta-cell transcription factor-positive or alpha-cell transcripition factor-negative glucagon-positive cells are involved. Thorel et al. concluded that alpha cells express beta-cell transcription factors, such as NKX6.1- and PDX-1, and convert to insulin-positive cells after beta-cell ablation^13^. On the other hand, in the study of Spijker et al.^15^ or Talchai et al.^27^, PDX-1-positive glucagon-positive cells are thought in the process of beta to alpha cells. According to the former report, the change in expression patterns of transcription factors in glucagon-positive cells is a compensatory reaction for the decreased beta-cell volume, and according to the latter reports, that is a cause of the decrease of beta cell^1,2^ and the increase of alpha cell in diabetes^16^. Increased expression of NKX6.1 in glucagon-positive insulin-negative cells, which indicates the loss of beta-cell identity, is attributed to beta-cell decrease in type 2 diabetes^24^, or centrally to an increase of alpha- to beta-cell conversion in type 1 diabetes^25^. It still remains to be investigated whether such cells constitute compensation for the reduction of beta-cell volume or are the cause of it, and the significance of our results is unknown. However, it is thought to be worthy to report that the relationships between the changes in expression patterns of islet-cell transcription factors and the decrease of beta-cell volume.

In conclusion, increased NKX6.1 expression and decreased ARX expression in glucagon-positive alpha cells closely correlate with the reduction of beta-cell volume in human pancreas.

## Research design and methods

### Patients

We enrolled Japanese patients who had undergone pancreatectomy between 2008 and 2013 at the Department of Gastroenterological Surgery, Osaka University Hospital, and had agreed to participate in this study. The study protocol was approved by the Ethics Committee of Osaka University (approval number: 13279-4). Patients with renal failure, pancreatic endocrine tumors, or who had been treated with antidiabetic agents were excluded from the study. Patients underwent a 75-g oral glucose tolerance test at 1–60 days before pancreatectomy, and were classified into three groups, normal glucose tolerance (NGT), impaired glucose tolerance (IGT), and type 2 diabetes mellitus (DM), in accordance with Japanese criteria^28^. Finally, 34 patients were enrolled. These patients were also included in our previous study^16^.

### Laboratory tests

We evaluated HbA1c, fasting plasma glucose (FPG), fasting C-peptide immunoreactivity (F-CPR), and increment of C-peptide by glucagon test (ΔC-peptide). CPR was measured by chemiluminescent enzyme immunoassay. The value of ∆C-peptide was defined as an increment in serum C-peptide level (nmol/L) at 6 min after intravenous injection of 1 mg glucagon after an overnight fast. We could not perform glucagon test for 15 patients because the inspection schedule could not be secured before operation. These data were obtained at 1–60 days before pancreatectomy.

### Pancreatic tissue processing

We obtained pancreas tissue samples from patients who underwent pancreatectomy. Pancreatic samples at normal region were collected during operation. The tissues were isolated from near the resected margins after intraoperative consultation, fixed immediately in formaldehyde, and embedded in paraffin for subsequent analysis. Paraffin-embedded tissues were cut into 5-μm-thick sections, stained with hematoxylin and eosin (HE), and confirmed to contain no cancerous elements. Sections with > 30% fibrous area as estimated by Azan staining were excluded from this study^16,29^.

### Immunohistochemistry

The primary and secondary antibodies as well as chromogenic substrates used are listed in the Supplemental Table. To evaluate the insulin-positive ratio of glucagon-positive cells (INS^+^/GCG^+^ ratio), NKX6.1-positive ratios of glucagon-positive cells (NKX6.1^+^/GCG^+^ ratios), and ARX-negative ratios of glucagon-positive cells (ARX^−^/GCG^+^ ratios), we performed double-immunofluorescent staining and counted these cells under a fluorescence microscope (BX53; Olympus, Tokyo, Japan). Heat-induced epitope retrieval (125 °C, 1 min) was performed in Target Retrieval Solution (Code No.: S1700; DAKO Japan, Kyoto, Japan). Pancreatic sections were incubated with anti-NKX6.1 or anti-ARX immunoglobulins as primary antibodies and biotinylated immunoglobulins as secondary antibodies, followed by streptavidin (Alexa Fluor 488-conjugated). Sections were then incubated with anti-glucagon or insulin immunoglobulins, followed by Alexa594- or rhodamine-conjugated immunoglobulins.

We examined one section per patient basically, as was shown our previous reports^16,29^, and two sections were examined in patients with small number (approximately fewer than 100) of glucagon-positive cells in one section. The procedure for measuring alpha- and beta-cell masses is also the same as in our previous papers^16,29^. As a surrogate for alpha-cell mass, we evaluated relative alpha-cell area and relative beta-cell area, which were determined by the proportion of glucagon-positive or insulin-positive cell area relative to the whole pancreatic section (%). Pancreatic sections were stained by the indirect immunoperoxidase method to measure the relative beta-cell area. Mouse anti-glucagon or guinea pig anti-insulin immunoglobulins were used as the primary antibodies, and biotinylated immunoglobulins were used as the secondary antibodies. The reactions were developed with an avidin–biotin complex and a 3,3-diaminobenzidine tetrahydrochloride substrate kit, followed by methyl green counterstaining. The areas of glucagon- and insulin-positive cells in the entire pancreatic section were quantified digitally with the WinROOF software program (Mitani Corporation, Fukui, Japan).

### Statistical analysis

Normally distributed data were compared by one-way analysis of variance followed by a post hoc Tukey–Kramer analysis. Multiple regression analyses were conducted to identify explanatory variable for the NKX6.1^+^/GCG^+^ ratio, and he F and p values were used in multiple regression analyses. P-value less than 0.05 and F value more than 4.0 were considered to denote a statistically significant difference. All statistical analyses were performed with JMP Pro 14 software (Statistical Analysis System Inc., Cary, NC, USA).

## Supplementary Information

Supplementary Information 1.

Supplementary Information 2.

## Data Availability

The data sets generated during this study are available from the corresponding author upon reasonable request.

## Abbreviations

ARX: Aristaless-related homeobox
ARX^−^/GCG^+^ ratio: Aristaless-related homeobox-negative ratio of glucagon-positive cells
α/β: Alpha- to beta-cell area ratio
ΔC-peptide: Increment of C-peptide by glucagon test
DM: Diabetes mellitus
F-CPR: Fasting C-peptide immunoreactivity
FPG: Fasting plasma glucose
IGT: Impaired glucose tolerance
NGT: Normal glucose tolerance
NKX6.1: NK homeobox 6.1
NKX6.1^+^/GCG^+^ ratio: NK homeobox 6.1-positive ratio of glucagon-positive cells
