# Supplementary figures and images for: Increased NKX6.1 expression and decreased ARX expression in alpha cells accompany reduced beta-cell volume in human subjects
Source: Sci Rep. 2021 Sep 7;11:17796. doi: 10.1038/s41598-021-97235-1 (PMC8423790; doi:10.1038/s41598-021-97235-1)

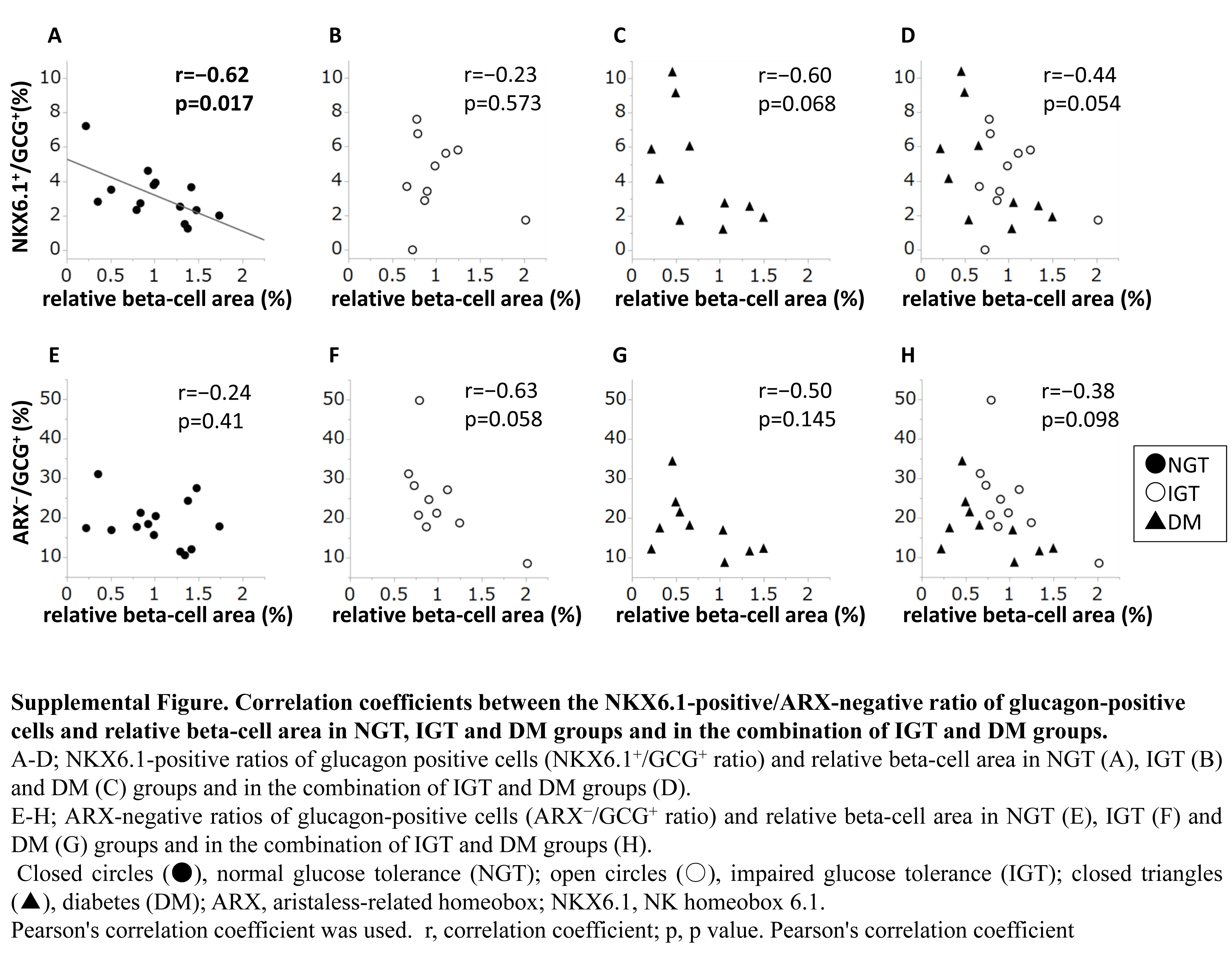

Supplement: Supplementary file 1 — Supplementary Information 1. [file 41598_2021_97235_MOESM1_ESM.tif]
