# Supplementary material for: Increased NKX6.1 expression and decreased ARX expression in alpha cells accompany reduced beta-cell volume in human subjects
Source: Sci Rep. 2021 Sep 7;11:17796. doi: 10.1038/s41598-021-97235-1 (PMC8423790; doi:10.1038/s41598-021-97235-1)
Supplement: Supplementary file 2 — Supplementary Information 2. [file 41598_2021_97235_MOESM2_ESM.docx]

Supplemental Table. Primary antibodies (A), secondary antibodies (B), and chromogenic substrates (C) used in this study.

A. Primary antibodies

| Antigen | Species | Source | RRID | Dilution |
| --- | --- | --- | --- | --- |
| Glucagon | Mouse | Sigma-Aldrich, MO, USA | AB_259852 | 1:500 |
| Glucagon | Rabbit | ImmunoStar, WI, USA | AB_572241 | 1:1000 |
| Glucagon | Guinea pig | Takara Bio Inc., Kusatsu, Japan | AB_2619627 | 1:500 |
| Insulin | Guinea pig | Dako Japan, Kyoto, Japan | AB_10013624 | 1:1000 |
| Somatostatin | Rabbit | Dako Japan, Kyoto, Japan | AB_2688022 | 1:200 |
| Pancreatic polypeptide | Rabbit | Abcam, Cambridge, UK | AB_11156699 | 1:1000 |
| NKX6.1 | Rabbit | Sigma-Aldrich, MO, USA | AB_10673664 | 1:50 |
| ARX | Mouse | [Merck Millipore](http://www.merckmillipore.com/JP/ja), Darmstadt, Germany | AB_11205581 | 1:50 |

NKX6.1, NK6 homeobox 1; ARX, aristaless-related homeobox

B. Secondary antibodies

| Antigen | Species | Source | RRID | Dilution |
| --- | --- | --- | --- | --- |
| Mouse | Horse (biotinylated) | Vector Laboratories, CA, USA | [AB_2313581](http://antibodyregistry.org/AB_2313581) | 1:200 |
| Mouse | Goat (Alexa Fluor 594-conjugated) | Molecular Probes, OR, USA | AB_141372 | 1:200 |
| Rabbit | Goat (biotinylated) | Vector Laboratories, CA, USA | AB_2313606 | 1:200 |
| Rabbit | Goat (Alexa Fluor 594-conjugated) | Molecular Probes, OR, USA | AB_2534095 | 1:200 |
| Guinea pig | Goat (biotinylated) | Vector Laboratories, CA, USA | AB_233613 | 1:200 |
| Guinea pig | Goat (Alexa Fluor 647-conjugated) | Abcam, Cambridge, UK | AB_2827756 | 1:200 |
| Guinea pig | Goat (rhodamine-conjugated) | Chemicon International, CA, USA | AB_2337428 | 1:200 |

C. Chromogenic substrates

| Chromogenic substrates | Source | RRID | Dilution |
| --- | --- | --- | --- |
| Avidin–biotin complex kit | Vector Laboratories, CA, USA |  |  |
| 3,3-diaminobenzidine | Molecular Probes, OR, USA |  |  |
| Streptavidin (Alexa Fluor 488-conjugated) | Life Technologies, CA, USA | AB_2315383 | 1:200 |
| Streptavidin (Alexa Fluor 647-conjugated) | Life Technologies, CA, USA |  | 1:200 |
